# Use of multiple micronutrient supplementation integrated into routine antenatal care: A discussion of research priorities

**DOI:** 10.1111/mcn.13722

**Published:** 2024-10-02

**Authors:** Tabassum Firoz, Jahnavi Daru, Jennifer Busch‐Hallen, Özge Tunçalp, Lisa M. Rogers

**Affiliations:** ^1^ Yale New Haven Health New Haven Connecticut USA; ^2^ Wolfson Institute for Population Health Queen Mary University of London London UK; ^3^ Global Technical Services Nutrition International Ottawa Ontario Canada; ^4^ Department of Sexual and Reproductive Health and Research including UNDP/UNFPA/UNICEF/WHO/World Bank Special Programme of Research, Development and Research Training in Human Reproduction (HRP) World Health Organization (WHO) Geneva Switzerland; ^5^ Department of Nutrition and Food Safety World Health Organization (WHO) Geneva Switzerland

**Keywords:** maternal nutrition, multiple micronutrient supplementation

## Abstract

Optimal maternal nutrition, including adequate intake and status of essential micronutrients, is important for the health of women and developing infants. Currently, the World Health Organization (WHO) *Antenatal care recommendations for a positive pregnancy experience* recommend daily iron and folic acid (IFA) supplementation as the standard of care. The use of multiple micronutrient supplements (MMS) is recommended in the context of rigorous research as more evidence was needed regarding the impact of switching from IFA supplements to MMS, including evaluation of critical clinical maternal and perinatal outcomes, acceptability, feasibility, sustainability, equity and cost‐effectiveness. WHO convened a technical consultation of key stakeholders to discuss research priorities with the objective of providing guidance and clarity to donors, implementers and researchers about this recommendation. The overarching principles of the research agenda include the use of clinical indicators and impact measures that are applicable across studies and settings and the inclusion of outcomes that are important to women. Future studies should consider using standardized protocols based on current best practices to measure critical outcomes such as gestational age (GA) and birthweight (BW) in studies. As GA and BW are influenced by multiple factors, more research is needed to understand the biological impact pathways, and how initiation and considerations for timing of MMS influence these outcomes. A set of core clinical indicators was agreed upon during the technical consultation. For implementation research, the Evidence‐to‐Decision framework was used as a resource for discussing components of implementation research. The implementation research questions, key indicators and performance measures will depend on country‐specific context and bottlenecks that require further research and improved solutions to enable the successful implementation of iron‐containing supplements.

## Key messages

- WHO recommends the use of MMS within the context of rigorous research. More evidence is desired on the impact of switching from supplements containing iron and folic acid alone to MMS, including evaluation of maternal and perinatal outcomes, acceptability, feasibility, sustainability, equity and cost‐effectiveness.
- Future research on MMS should include clinical indicators and impact measures that are applicable across studies and settings, and incorporate outcomes important to women, using standardized protocols.
- Specific research questions, key indicators and performance measures of implementation research activities will depend on the country context. This research can help improve the effectiveness of supplementation programmes and strengthen antenatal care services.

## Graphical abstract

World Health Organization recommends iron and folic acid supplementation in pregnancy and has called for additional clinical and implementation research on the use of multiple micronutrient supplements. Research priorities include the use of clinical indicators applicable across studies, standardized protocols to measure critical outcomes and country‐specific implementation research questions and performance measures.

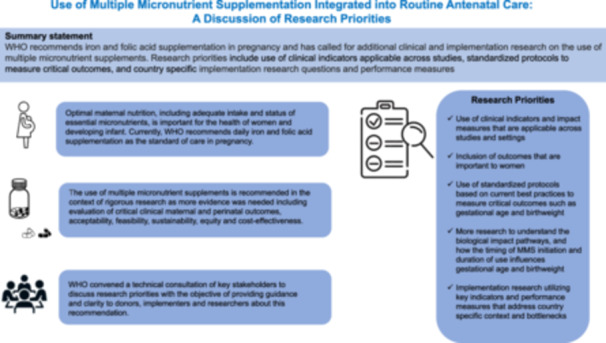

## 1 INTRODUCTION

Optimal maternal nutrition, including adequate intake and status of essential vitamins and minerals, is important for maternal health and fetal development. A recent pooled analysis estimated that the global prevalence of deficiency in at least one of three micronutrients is about 69% among nonpregnant women 15–49 years of age, and this prevalence is likely higher in pregnant women due to their increased micronutrient requirements (Stevens et al., 2022).

The *WHO antenatal care recommendations for a positive pregnancy experience* recommend daily iron and folic acid (IFA) supplements as the standard of care as part of routine antenatal care (ANC) (World Health Organization [WHO], 2016c). In 2020, the recommendation for multiple micronutrient supplements (MMS) in pregnancy was updated from ‘not recommended’ to ‘recommended in the context of rigorous research’ (WHO, 2020). Although the evidence suggested that there may be limited benefit and possibly little harm in replacing IFA supplements with MMS, two critical research implications, one clinical and one from implementation, underline this updated recommendation.

With regard to clinical research, the WHO Guideline Development Group (GDG) found the evidence on low birthweight and its component parts (preterm birth and small for gestational age [SGA]) difficult to interpret and additional clinical evidence was called for. Regarding implementation research, it was recommended that further evidence is needed to establish the impact of switching from IFA supplements to MMS, including evaluation of acceptability, feasibility, sustainability, equity and cost‐effectiveness within a given country programme (WHO, 2020). While WHO currently does not recommend switching from the use of IFA to MMS in routine ANC, if a country is considering a switch from IFA to MMS, it is recommended that this be done within the context of implementation research.

In September 2022, WHO convened a technical consultation of a diverse group of key stakeholders (such as funders, nongovernmental organizations, international maternal and newborn health organizations, clinicians, scientists and researchers) to discuss research priorities to address these two critical areas. The purpose of the technical consultation was to unpack the current guideline recommendation to identify what clinical and implementation research would be most beneficial for addressing the questions posed by the GDG. As this was not a consensus meeting, evidence was discussed but not evaluated.

This paper summarizes the discussions during the consultation with the aim to better guide donors, implementers and researchers on a way forward.

## 2 CONTEXT OF THE UPDATED RECOMMENDATION ON THE PROVISION OF MMS DURING PREGNANCY

In 2016, an analysis of the evidence for the guideline development process suggested that compared to IFA supplements, MMS might lead to a reduction in the rates of low birthweight babies, but the lack of other demonstrable benefits (e.g. further reduction of anaemia prevalence), equivocal evidence on neonatal mortality related to the dose of iron (30 or 60 mg) used, the higher cost of MMS and concerns about feasibility led the GDG to not recommend MMS for all pregnant women as part of routine ANC (Tuncalp et al., 2020). The GDG noted that policymakers in populations with a high prevalence of nutritional deficiencies might consider the benefits of MMS to outweigh the disadvantages and may choose to give them to pregnant women instead of IFA supplements only (WHO, 2020).

Following the living guideline approach used for maternal and perinatal health recommendations (Vogel et al., 2019), additional trials and an individual participant data meta‐analysis published after the release of the 2016 ANC guideline led the WHO to review the previous recommendation on MMS. In 2020, a review of the evidence on effectiveness was found to be largely similar to that evaluated during the 2016 guideline development process. There was an average 12% (9%–14%) reduction in low birthweight with MMS but little difference when individually looking at the two component parts of low birthweight (preterm birth or SGA) (WHO, 2020). The evidence also showed that switching from IFA supplements may be cost‐effective in some countries and relatively favourable equity, acceptability and feasibility. These considerations led to a decision to recommend MMS in the context of rigorous research (WHO, 2020) (Box 1).

Box 1 **Research priorities identified by the GDG in 2020**

1

1. Impact of switching to a 30 mg dose of iron (as provided in MMS) from a higher dose of iron (e.g. 60 mg, as commonly provided in IFA), particularly in settings where higher doses of iron are routinely used due to high anaemia prevalence or other reasons.
2. Impact of IFA and MMS in controlled clinical trials using early pregnancy ultrasound to establish gestational age with certainty, with assessment of critical maternal and perinatal outcomes and follow‐up of infants sustained into childhood.

Abbreviations: GDG, World Health Organization's Guideline Development Group; IFA, iron and folic acid; MMS, multiple micronutrient supplement.

## 3 DEFINING RIGOROUS RESEARCH

Within the WHO guideline for routine ANC, the GDG has the option to (1) recommend the intervention, (2) not recommend the intervention or (3) recommend the intervention only under certain conditions (e.g. in specific contexts, targeted monitoring and evaluation or rigorous research) (WHO, 2016c). A context‐specific recommendation ‘only in the context of rigorous research’ was made for MMS in 2020. For the purposes of MMS, this recommendation includes clinical and implementation research. Other WHO recommendations that are recommended in the context of rigorous research include the following: zinc supplementation, daily fetal movement counting, antibiotics for recurrent urinary tract infections and anti‐D immunoglobulin in non‐sensitized women.

## 4 CLINICAL RESEARCH

Reanalyses of existing trial data, as well as a small number of new trials, have emerged since the updated recommendation in 2020 (Box 2). One of these reanalyses (discussed below) aimed to address the question of assessment of gestational age (Gomes et al., 2023) while a new trial is planned to determine the optimal dose of iron. Given the research priorities recommended by the GDG, some participants of the technical consultation felt there was a need for better standardization and harmonization of the measurement, definition and interpretation of critical clinical maternal and perinatal outcomes, not only for data collection in future studies but also when conducting reanalyses. The participants of the technical consultation recognized that new efficacy trials using the same micronutrient formulations and outcomes are unlikely to alter the overall effect size on birthweight and gestational age. However, future trials on any micronutrient supplement may use gestational age and birthweight as critical maternal and perinatal outcomes, in addition to changes (positive and negative) on biomarkers associated with maternal micronutrient status, as well as those related to infection and/or inflammation. Standardizing or harmonizing these clinical indicators and outcomes will ensure applicability across studies, allowing for meaningful comparison.

### 4.1 Key research questions

#### 4.1.1 Assessment of gestational age

In the 2020 update, gestational age emerged as an important area to address in future trials. The GDG recommended that controlled clinical trials use early pregnancy ultrasound to establish gestational age with certainty to improve the accuracy of perinatal outcome assessments (WHO, 2020).

Existing studies have used a variety of methods to assess gestational age, including last menstrual period (LMP) alone, LMP with urine pregnancy test and ultrasound. However, it is estimated that up to 30% of women will not have a reliable LMP (Napolitano et al., 2014).

A new meta‐analysis looked at the effect of IFA compared to MMS by the method of gestational age estimation (Gomes et al., 2023). This meta‐analysis utilized data from the 16 trials that contributed to the outcomes of low birthweight, preterm and SGA in the WHO GDG analysis; seven trials (44%) assessed gestational age by ultrasound, four trials (25%) used a prospective collection of LMP and pregnancy urine test (a validated method) and five trials (31%) used recall of date of LMP. The meta‐analysis suggests the effect of MMS compared to IFA on three birth outcomes (low birthweight, preterm birth and SGA) did not vary significantly across the three methods of gestational age estimation (Gomes et al., 2023). When limited to the seven trials that used ultrasound, the magnitude of the beneficial effect of MMS was higher for all birth outcomes, including low birthweight (relative risk [RR]: 0.87; 95% confidence interval [CI]: 0.78–0.97), preterm birth (RR: 0.90; 95% CI: 0.79–1.03) and SGA (RR: 0.90; 95% CI: 0.83–0.99) (Gomes et al., 2023).

Where ultrasound was used in these studies, the timing of use varied between 9 and 17 weeks (WHO, 2020). The measurement of fetal crown‐rump length (CRL) by ultrasound in the first trimester (before 14 weeks gestation) is the recommended standard method for pregnancy dating (Napolitano et al., 2014; Noguchi et al., 2023; Papageorghiou et al., 2016). Some participants recognized that there may be challenges with early gestational age assessment in routine clinical care. WHO recommends one ultrasound scan before 24 weeks of gestation for pregnant women to estimate gestational age, improve detection of fetal anomalies and multiple pregnancies, reduce induction of labour for post‐term pregnancy and improve a woman's pregnancy experience (Noguchi et al., 2023).

In order for the results of birth outcomes to be easily interpretable across research studies, consideration should be given to developing a standardized protocol for future trials to unify ultrasound technique, timing, data entry and outcome assessment. The group recognized several potential challenges to achieving this, such as recruiting women to attend ANC in the first trimester, trained staff and access to maintained and accurate ultrasound machines.

#### 4.1.2 Assessment of birthweight

The WHO GDG analysis found a 12% reduction in low birthweight when MMS was compared to IFA supplements (WHO, 2020). However, the panel had difficulty interpreting the clinical significance of this finding because it reflects the number of babies born preterm plus the number of babies born at term that is defined as SGA, for which the evidence suggested no difference in effect between MMS and IFA supplements (WHO, 2020). Therefore, another key area of research emerging during the consultation is optimal birthweight measurement and consistent reporting across studies.

In the existing literature, variations in birthweight assessment were multi‐factorial, including scale calibration, repeated birthweight assessment and timing of measurement after birth. Studies included in the GDG analysis varied substantially on the timing of the assessment of birthweight. While some studies measured birthweight ‘as soon as possible after birth’, others measured birthweight within 1 h, 24 h, 48 h, 72 h and even up to 14 days after birth. The two (out of 19 trials) that assessed some weights up to 14 days after birth (to avoid missing data) attempted to address this by back‐calculating the weight by using formulae described by the WHO (Adu‐Afarwuah et al., 2015; Ashorn et al., 2015).

The variation in the timing of birthweight measurement is important to standardize within and between future trials as infants lose 7%–8% of their birthweight within the first week of life (DiTomasso & Cloud, 2019). A standardized approach based on current best practices could include guidance on timing and frequency of birthweight measurement, along with proper calibration of scales. Some participants felt that regional differences in birthweight were an important consideration, and birthweight centiles may be useful as they factor in the impact of ethnicity, gestational age and maternal height and weight on infant birthweight and estimated fetal weight (Kiserud et al., 2017).

#### 4.1.3 Optimal dose of iron

The WHO GDG highlighted the need for more evidence on the effects of switching to 30 mg of elemental iron from a higher dose (e.g. 60 mg). This is particularly relevant in areas where there is a high burden of maternal anaemia. To address this, data from a meta‐analysis of 11 trials comparing MMS vs IFA containing either 30 or 60 mg of elemental iron was presented (Gomes et al., 2022a). There were five trials where MMS containing 30 mg of iron was compared with IFA containing 60 mg of elemental iron. Within these trials, there were 4677 participants with a high mean baseline anaemia prevalence (between 27% and 49%) and results suggested there was no difference in third‐trimester anaemia rates (RR: 0.99; 95% CI: 0.92–1.07) (Gomes et al., 2022a). Analysis of seven trials with 14,144 participants looking at the same comparisons also did not show a statistically significant difference in neonatal mortality (risk ratio: 1.12; 95% CI: 0.83‐1.50) (Gomes et al., 2022b).

Additional primary data on the optimal iron dose for use in MMS and IFA supplements would be valuable. There is a planned superiority trial comparing MMS preparations containing 30, 45 and 60 mg of elemental iron among pregnant women in Tanzania with a sample size of approximately 6000 women and maternal haemoglobin measured in the third trimester will be reported (National Library of Medicine U.S., 2024).

### 4.2 Additional research questions

#### 4.2.1 Composition, bioavailability and risk of intake above tolerable upper limits of micronutrients

Several meeting participants noted that the optimal micronutrient composition and dose of each vitamin and mineral comprising MMS may need further study while recognizing that it is a complex research agenda. The UN International Multiple Micronutrient Antenatal Preparation (UNIMMAP) is the most commonly used formulation. This formulation was developed as a result of a consultation held by UNICEF, WHO and the United Nations University in 1999 for use in trials designed to assess the benefits or risks of replacing IFA supplements (World Health Organization, United Nations University & United Nations Children's Fund ‎UNICEF, 1999).

Nutrient deficiencies vary considerably across countries, being driven by numerous factors, including dietary preferences, food availability and affordability and national fortification strategies, to name a few. A review of Recommended Nutrient Intake (RNI) values has suggested that limited primary data are available to understand micronutrient requirements in pregnancy (Smith et al., 2021). These values are designed to meet the requirements of almost all apparently healthy individuals in an age‐, sex‐ and physiologic‐specific population group rather than treat nutritional deficiencies. Data from the largest MMS trial in Bangladesh showed that MMS reduced adverse birth outcomes but was unable to eliminate existing deficiencies and did not further reduce anaemia prevalence or prevent more women from falling into a deficient status by the third trimester (Schulze et al., 2019). There are planned trials in Bangladesh and Tanzania that will assess different doses of multiple micronutrients in pregnant women. A future direction can also include studying the role of micronutrients in relationship to the underlying cause of anaemia.

There was also discussion on the risk of reaching micronutrient intakes above the tolerable upper limit with the use of MMS in conjunction with other micronutrient interventions. A recent commentary has raised concern about the risks of layering interventions containing the same micronutrients (e.g. mandatory micronutrient fortification with iodine, vitamin A and zinc, distribution of fortified supplementary foods with multiple micronutrients and MMS containing these same micronutrients) (Kurpad & Sachdev, 2022). While an existing publication has found that MMS using the UNIMMAP formulation in addition to a nutritionally adequate diet (i.e. already including the recommended intake of the 15 micronutrients) does not result in excessive micronutrient intakes (Gernand, 2019) the presumption of no harm needs to be examined for any supplement especially if there is layering of interventions for the same micronutrient (e.g. diet, fortification and supplementation).

It has been proposed that there needs to be a move towards precision in nutrition prescription which includes individualized micronutrient needs assessment and based on accurate use of nutritional biomarkers. However, at present, there is a paucity of metabolic or pharmacokinetic studies on MMS. While the group did not reach a consensus on specific biomarker use, some potential micronutrients proposed for the study include iron, folate, vitamin B12, vitamin A, vitamin D and iodine.

The participants were fully supportive of the WHO recommendation that pregnant women should be supported and encouraged to receive adequate nutrition through the consumption of a healthy, balanced diet. There was also discussion that future research on MMS should include an intervention arm aimed at improving dietary diversification.

#### 4.2.2 Biological impact pathways

Biological mechanisms underlying the effects of MMS on pregnancy outcomes are not well elucidated and there is a complex interplay of multiple micronutrients in various stages of fetal development. Preterm birth and growth restriction could be the endpoints of different pathways and a recent review outlines how undernourishment, infection, psychological stress and environmental exposures have the potential to act on both pathways through intermediates of oxidative stress, inflammation, inadequate immune protection and placental dysfunction (Ashorn et al., 2023).

While it is unlikely that a better understanding of the biological impact pathways would change the evidence profile of MMS, some participants of the technical consultation felt that more research is needed as it could influence the composition of MMS and the timing of administration. As part of this technical meeting, we searched and assessed all of the published literature for possible biological impact pathways. Most published studies in this area are secondary analyses of larger trials with limitations, rather than primary mechanistic work. While several mechanisms of biological impact were explored, the results of these studies are inconclusive for the most part (Table S1).

One potential mechanism that raised interest among the group was the Hypertensive Disorders of Pregnancy, particularly preeclampsia, which is associated with and commonly a causal factor in both preterm birth and low birthweight. Preeclampsia is a complex condition that results in abnormal placentation with inflammation, oxidative stress and blood pressure being key components in its development (Kinshella et al., 2023). Nutritional risk factors interact with all other risk factors involved in preeclampsia, such as genetic, clinical, social and environmental risk factors. A recent evidence review showed that some dietary factors, especially calcium, vitamin D, iron and overall maternal diet, appear to affect preeclampsia rates (Kinshella et al., 2023). Many of the mechanisms underlying the development of preeclampsia have been studied in the limited body of literature on MMS impact pathways, as outlined in Table S1. As such, many members of the group felt that it would be reasonable to include preeclampsia as a critical maternal outcome, bearing in mind that preeclampsia is an evolving area of research.

#### 4.2.3 Timing of initiation of MMS

Timing and duration of MMS are important considerations to determine the optimal exposure window for nutritional interventions. The first trimester particularly encompasses several critical gestational processes, including placentation which is implicated in the biological mechanism of preeclampsia. In the current body of evidence, gestational age at enrolment and, therefore, initiation of MMS ranged from 9 to 29 weeks (Gomes et al., 2023). Whether the timing of initiation of MMS influences birth outcomes is unknown, and as such, analyses of existing data, as well as future studies, can explore birth outcomes by the timing of initiation.

### 4.3 Key clinical indicators

The following key clinical indicators were prioritized as beneficial for study investigators to consider in future research on micronutrient supplements, including MMS (Table 1).

**Table 1:** Key clinical indicators.

Indicator	Comments
Maternal	
Maternal nutritional status	Assessment of baseline nutritional status (e.g. using a validated nutrition checklist such as the FIGO Nutrition Checklist) (Killeen et al., 2023) to improve understanding of underlying deficiencies (e.g. iron, folate, vitamin B12, vitamin A, vitamin D, iodine) and factors influencing multiple indicators (e.g. maternal anaemia, inflammation and infection).
Maternal anaemia	Serial haemoglobin measurements (e.g. three over time) to better understand the impact of an intervention. Assess determinants of anaemia to better characterize the underlying cause of anaemia (e.g. nutritional deficiency, infection, inherited red blood cell disorder). Evaluate regional‐specific causes of nutritional deficiencies.
Maternal comorbidities	Assessment of pre‐existing maternal conditions (e.g. chronic hypertension, diabetes, etc.) to improve understanding of how MMS impacts maternal health overall, in addition to improving understanding of how MMS impacts birthweight.
Placental phenotypes	History of hypertensive disorders of pregnancy, gestational diabetes, other placental syndromes (such as placental abruption and stillbirth).
Access to ANC	Participant access and availability to health care, and specifically ANC, to improve capacity building and understanding of how services can be adapted.
Adherence and duration of supplementation	Formulation of MMS, time of initiation, frequency and duration of use to understand participant compliance and uptake of MMS.
Women reported outcomes	Acceptability of MMS. Side effects/adverse events (e.g. constipation, diarrhoea, nausea, vomiting). Experience of care (Mehrtash, Stein, Barreix, Bonet, et al., 2023).
Perinatal	
Gestational age	Measurement of gestational age, estimated using ultrasound between 9^+0^ and 13^+6^ weeks (Papageorghiou et al., 2014) reported as a continuous variable, recognizing that term pregnancy will influence the upper limit of this indicator. Define the term pregnancy. For example, using the ICD‐10 definition (WHO, 2016a).
Birthweight	Measurement of birthweight using electronic scales calibrated twice a week at minimum, and measured by two individuals independently within 12 h of birth (de Onis et al., 2012) and reported as a continuous variable. Use of birthweight centiles.
Longer term perinatal and childhood outcomes	Assessment of long‐term outcomes (e.g. growth at 3 months, failure to thrive at 6–12 months, childhood stunting) to estimate whether the observed outcomes are sustained beyond the initial neonatal period.

Abbreviations: ANC, antenatal care; FIGO, International Federation of Gynecology and Obstetrics; ICD‐10, 10th revision of the International Classification of Diseases; MMS, multiple micronutrient supplement.

In 2002, a meeting of researchers and principal investigators of multiple micronutrient trials, and policy‐makers, funded by the Micronutrient Initiative, agreed on a few core indicators measured in a standardized fashion to ensure comparability across various trials and to maximize possibilities of pooling data across sites (UNICEF, UNU, WHO Study Team, 2002). However, a future goal would be to build upon this core set of outcome indicators that represents a minimum data set and develop robust methods through engagement with diverse stakeholders, including health care professionals, researchers and women (Duffy et al., 2020).

## 5 IMPLEMENTATION RESEARCH

Some country programmes have already, or are in the process of, considering a switch to MMS from IFA. In these cases, the 2020 WHO guidance recommends for such a switch to occur in the context of implementation research.

### 5.1 What is the current landscape of MMS implementation research?

As part of the technical consultation, a landscape analysis of current and planned implementation research was conducted. This involved discussions, interviews and reviews of programmatic and research activity carried out by governmental and nongovernmental organizations and UN agencies across the world.

This analysis indicated that a variety of activities are ongoing, ranging in approach and complexity. Some groups focused their activities on ensuring coverage of MMS was maintained through working with dispensing teams and providing access to MMS outside of ANC settings, while other activities were focused on embedding MMS into existing national ANC platforms.

Box 2 provides a summary of the types of ongoing and planned implementation research projects and programmatic activities in countries that are considering a switch to MMS.

Box 2 **Summary of the types of ongoing implementation research projects and programmatic activities**

1

1. There are several global and national projects directed at increasing the availability of MMS through existing ANC platforms, maternity care providers (e.g. home visits) and other health care entry points (e.g. pharmacy dispensing or purchasing options) from a variety of governmental and nongovernmental providers.
2. There are national programmes assessing the uptake and adherence of MMS in areas where IFA is routinely used. This includes expanding programmes using the infrastructure of existing academic projects of MMS with this as an additional aim.
3. There are several smaller specific nongovernmental organizations and charities focusing on the importing, consignment testing, warehousing and distribution of MMS in areas where antenatal health programmes are more fragmented
4. Programmes assessing the cost‐benefit analysis of switching to MMS from IFA at regional and national levels with partnerships across academia and other stakeholders.
5. There are other public health programmes where the focus remains on developing local production of MMS. This may include procurement of raw ingredients for MMS production, quality assurance and regulatory implications, in nations where MMS is considered a medicinal product.

Abbreviations: ANC, antenatal care; IFA, iron and folic acid; MMS, multiple micronutrient supplement.

### 5.2 What is implementation research within MMS programmes?

Implementation research offers an opportunity to improve programme effectiveness and shines a much‐needed light on how to strengthen ANC programmes towards achieving a positive pregnancy experience. Implementation research can help inform decision‐making around the integration of iron‐containing supplements (whether IFA or MMS) into the existing health system through the ANC service delivery platform where there is not already integration. As MMS is a relatively new commodity, small‐scale pilot programmes are often implemented within an ANC platform where the switch from IFA to MMS is being considered.

It is important to recognize that implementation research requires an investment in both time and finances and requires planning and involvement of external stakeholders (e.g. nutritionists, clinicians and implementation scientists) in addition to programme staff. Participatory research in the context of MMS has been largely limited to the assessment of its acceptability by women. WHO maternal health recommendations prioritize person‐centred health and well‐being, which more comprehensively includes maternal competence and autonomy (Mehrtash, Stein, Barreix, Bohren, et al., 2023). In keeping with these principles, some participants felt it was important to ensure that both future clinical and implementation research considers outcome measures that relate to experience of care.

Appreciating the ongoing challenges of carrying out research in real‐life settings in LMICs, identifying a support network of those in a similar situation may be imperative for overcoming hurdles. Similarly, ensuring that any learnings and findings from existing implementation research programmes are disseminated will be instrumental in evolving policy in this area.

Box 3 describes a process that can be adapted for undertaking implementation research. The domains of the Evidence‐to‐Decision (EtD) framework (WHO, 2016b) served as a resource to facilitate discussion on benefits and harms, acceptability, feasibility, equity, resources and sustainability. Although not every step will be applicable to all in all contexts, these steps can be used as a general approach for research planning and project development.

Box 3 **Considerations for undertaking implementation research for MMS**

1

1. Enable informed government decision‐making and leadership in each step of the implementation research. Establish or utilize an existing government technical working/advisory group and use, if possible, existing expertise in programming and research.
2. Assess the landscape and the enabling environment. This may include considering the following areas:
  a. Understand the health, nutrition profile and anaemia burden among women and newborns.
  b. Assess the policy and strategy environment.
  c. Assess the strength and utilization of the existing ANC platform(s).
    i. Private and public, routine and campaign, health facilities and community.
  d. Study the current enablers and barriers to uptake of IFA (if it is currently being used) to gain insight that can used to inform future changes.
  e. Study the market (consider both the supply side and demand side).
    i. Supply (assess regulations, inclusion in essential medicines list, availability of locally produced MMS [UNIMAPP], potential local manufacturing for future supply, possible imports in the short‐term).
    ii. Demand (ANC utilization, forecasting, considering social norms and cultural and behavioural drivers).
  f. Understand and translate the global guidance (WHO ANC recommendations) and evidence for considering the transition to MMS to the country context.
  g. Understand the potential added benefits of MMS compared to IFA.
  h. Assess the cost–benefit of transitioning from IFA to MMS (e.g. using the *Nutrition International MMS cost–benefit tool*).

Strategies to assess and treat maternal anaemia, particularly in areas with an existing high prevalence, should be in place and aligned with current recommendations.

Possible benefits:

Increased attendance at ANC if a new product (MMS) is offered as part of the service.
Strengthened maternity health system with cross‐training across health care providers.
Opportunity to review other programmes in ANC services (e.g. number of visits, availability of dating scans).

Possible harms:

Overburdening a health care system by introducing a new policy and product.
Time and training requirements of health care providers at individual and institutional levels.
Possibility of unintended consequences (e.g. conflicts of interest):

3. Define what area of IR is the focus for the project (e.g. focus on procurement or local production). Consider additional health and nutrition benefits of transitioning to MMS.
4. Develop the IR research question(s) by conducting an objective process for identifying the priority questions for your country/region (e.g. using the *Child Health and Nutrition Research Initiative* [CHNRI] process). https://methods.cochrane.org/prioritysetting/blog/child-health-and-nutrition-research-initiative-chnri-approach-research-priority-setting
5. Introducing MMS into the ANC platform.
  a. Consider procurement, import (or local production of MMS) and labelling requirements along with warehousing.
  b. Consider quality control of products (e.g. storage temperature), quality assurance and capacity.
  c. Consider sustainability (e.g. whether the supply chain will be able to support scale‐up).
  d. Regular distribution to health facilities/Last mile distribution through the existing health system.
  e. Develop MMS programme tools, protocols and job aids (standard operating procedures, fact sheets, counselling cards, FAQs).
  f. Adapt monitoring tools and procedures to feed into existing systems where possible.
  g. Train health care providers and integrate MMS in supportive supervision.
  h. Develop clear guidance on MMS for the prevention of anaemia and support the continuation of existing diagnosis and treatment guidelines for anaemia, including IFA supplementation.
  i. Community engagement especially understanding women's preferences (IFA vs. MMS), expectations and knowledge.
  j. Ongoing monitoring and course correction.
6. Conduct the implementation research in collaboration with women, communities, health care providers, stakeholders, researchers and methodologists.
  a. Consider starting with a situation analysis, formative research, sex and gender‐based analysis and cultural and behavioural research.
  b. Improving adherence is a common research focus. Human‐centred design methodology can be key to finding adherence solutions.
  c. Evaluation: Depending on the research question, outcome, process and cost‐effectiveness, evaluations can be used to test the hypothesis and assess your implementation research outcomes of interest (acceptability, feasibility, equity, cost‐effectiveness, sustainability).
  d. Equity (e.g. consider issues like donations versus self‐pay, coverage, geography, seasonality) including studying disparities to access, particularly in LMICs.
7. Synthesis and translation of the research findings and programme learnings for policy, programme and financial decision‐making.

Abbreviations: ANC, antenatal care; FAQs, frequently asked questions; IFA, iron and folic acid; LMICs, low‐ and middle‐income countries; MMS, multiple micronutrient supplement.

## 6 CONCLUSION

This technical consultation was an effort to bring together stakeholders and experts from both the areas of nutrition and ANC to discuss research priorities that would move the agenda on the use of iron‐containing supplements during routine ANC forward in an aligned way. One of the critical outputs of the technical consultation was unpacking the updated 2020 WHO recommendation on the use of MMS in pregnancy within the context of rigorous research.

To address the WHO GDG's recommendation on accurate measurement of gestational age, it was proposed that a standardized approach using current best practices be used in future studies of nutritional supplements. As it is difficult to interpret the evidence on preterm birth and its component parts, a similar recommendation was made to standardize the measurement of birthweight. A set of critical maternal and perinatal clinical indicators that can be collected in future studies were suggested. A trial is underway to address the research question on the optimal dose of iron in MMS, while other research topics identified by the group included composition and bioavailability of micronutrients in MMS, biological impact pathways of MMS and potential for consumption of micronutrients in excess of the tolerable upper intake levels, particularly when MMS is provided along with other micronutrient interventions.

Clarification on what entails implementation research allowed the group to identify appropriate implementation research questions; however, it was agreed that key indicators and performance measures will depend on what phase of implementation a country is in. The domains of the EtD framework could be used a guide for programmatic evaluation in the context of MMS.

The outputs of this technical consultation can serve as a basis for standardizing and harmonizing future clinical and implementation research on the use of iron‐containing supplements during the antenatal period and help strengthen the evidence base for better‐informing programmes considering a switch from IFA to MMS.

## AUTHOR CONTRIBUTIONS

Tabassum Firoz drafted the manuscript with input from Jahnavi Daru and Jennifer Busch‐Hallen. All authors contributed equally to critically editing the manuscript and approving the final version.

## CONFLICT OF INTEREST STATEMENT

The authors declare no conflict of interest. A declaration of interest form was completed by all participants of the consultation and verbal declarations were made at the start of the consultation.

## Supporting information

Supporting information.

## Data Availability

Please forward requests for data to the corresponding author.

## References

[mcn13722-bib-0001] Adu‐Afarwuah, S. , Lartey, A. , Okronipa, H. , Ashorn, P. , Zeilani, M. , Peerson, J. M. , Arimond, M. , Vosti, S. , & Dewey, K. G. (2015). Lipid‐based nutrient supplement increases the birth size of infants of primiparous women in Ghana. The American Journal of Clinical Nutrition, 101(4), 835–846. 10.3945/ajcn.114.091546 25833980

[mcn13722-bib-0002] Ashorn, P. , Alho, L. , Ashorn, U. , Cheung, Y. B. , Dewey, K. G. , Gondwe, A. , Harjunmaa, U. , Lartey, A. , Phiri, N. , Phiri, T. E. , Vosti, S. A. , Zeilani, M. , & Maleta, K. (2015). Supplementation of maternal diets during pregnancy and for 6 months postpartum and infant diets thereafter with small‐quantity lipid‐based nutrient supplements does not promote child growth by 18 months of age in rural Malawi: A randomized controlled trial. The Journal of Nutrition, 145(6), 1345–1353. 10.3945/jn.114.207225 25926413

[mcn13722-bib-0003] Ashorn, P. , Ashorn, U. , Muthiani, Y. , Aboubaker, S. , Askari, S. , Bahl, R. , Black, R. E. , Dalmiya, N. , Duggan, C. P. , Hofmeyr, G. J. , Kennedy, S. H. , Klein, N. , Lawn, J. E. , Shiffman, J. , Simon, J. , Temmerman, M. , Krasevec, J. , Bradley, E. , Conkle, J. , … Hayashi, C. , UNICEF‐WHO Low Birthweight Estimates Group . (2023). Small vulnerable newborns‐big potential for impact. The Lancet, 401(10389), 1692–1706. 10.1016/S0140-6736(23)00354-9 37167991

[mcn13722-bib-0004] de Onis, M. , Cheikh Ismail, L. , Chumlea, C. , Onyango, A. , Bhutta, Z. , Luna, M. S. , & Knight, H. (2012). *INTERGROWTH‐21st: International fetal and newborn growth standards for the 21st century: Anthropometry handbook*. https://media.tghn.org/articles/Anthropometry_Handbook_April_2012.pdf

[mcn13722-bib-0005] DiTomasso, D. , & Cloud, M. (2019). Systematic review of expected weight changes after birth for full‐term, breastfed newborns. Journal of Obstetric, Gynecologic & Neonatal Nursing, 48(6), 593–603. 10.1016/j.jogn.2019.09.004 31610141

[mcn13722-bib-0006] Duffy, J. M. N. , Cairns, A. E. , Magee, L. A. , von Dadelszen, P. , van 't Hooft, J. , Gale, C. , Brown, M. , Chappell, L. C. , Grobman, W. A. , Fitzpatrick, R. , Karumanchi, S. A. , Lucas, D. N. , Mol, B. , Stark, M. , Thangaratinam, S. , Wilson, M. J. , Williamson, P. R. , Ziebland, S. , & McManus, R. J. , International Collaboration to Harmonise Outcomes for Pre‐eclampsia (iHOPE) . (2020). Standardising definitions for the pre‐eclampsia core outcome set: A consensus development study. Pregnancy Hypertension, 21, 208–217. 10.1016/j.preghy.2020.06.005 32674052

[mcn13722-bib-0007] Gernand, A. D. (2019). The upper level: Examining the risk of excess micronutrient intake in pregnancy from antenatal supplements. Annals of the New York Academy of Sciences, 1444(1), 22–34. 10.1111/nyas.14103 31094004 PMC6618111

[mcn13722-bib-0008] Gomes, F. , Agustina, R. , Black, R. E. , Christian, P. , Dewey, K. G. , Kraemer, K. , Shankar, A. H. , Smith, E. R. , Thorne‐Lyman, A. , Tumilowicz, A. , & Bourassa, M. W. (2022a). Multiple micronutrient supplements versus iron‐folic acid supplements and maternal anemia outcomes: An iron dose analysis. The Lancet Global Health, 1512(1), 114–125. 10.1111/nyas.14756 PMC9306935 35218047

[mcn13722-bib-0009] Gomes, F. , Agustina, R. , Black, R. E. , Christian, P. , Dewey, K. G. , Kraemer, K. , Shankar, A. H. , Smith, E. , Tumilowicz, A. , & Bourassa, M. W. (2022b). Effect of multiple micronutrient supplements vs iron and folic acid supplements on neonatal mortality: A reanalysis by iron dose. Public Health Nutrition, 25(8), 1–13. 10.1017/S1368980022001008 PMC9991737 35466910

[mcn13722-bib-0010] Gomes, F. , Askari, S. , Black, R. E. , Christian, P. , Dewey, K. G. , Mwangi, M. N. , Rana, Z. , Reed, S. , Shankar, A. H. , Smith, E. R. , & Tumilowicz, A. (2023). Antenatal multiple micronutrient supplements versus iron‐folic acid supplements and birth outcomes: Analysis by gestational age assessment method. Maternal & Child Nutrition, 19(3), e13509. 10.1111/mcn.13509 37002655 PMC10262881

[mcn13722-bib-0011] Killeen, S. L. , Donnellan, N. , O'reilly, S. L. , Hanson, M. A. , Rosser, M. L. , Medina, V. P. , Jacob, C. M. , Divakar, H. , Hod, M. , Poon, L. C. , Bergman, L. , O'brien, P. , Kapur, A. , Jacobsson, B. , Maxwell, C. V. , McIntyre, H. D. , Regan, L. , Algurjia, E. , Ma, R. C. , … The FIGO Committee on Impact of Pregnancy on Long‐term Health and the FIGO Division of Maternal and Newborn Health . (2023). Using FIGO nutrition checklist counselling in pregnancy: A review to support healthcare professionals. International Journal of Gynaecology and obstetrics: The Official Organ of the International Federation of Gynaecology and Obstetrics, 160(Suppl 1), 10–21. 10.1002/ijgo.14539 36635083 PMC10108324

[mcn13722-bib-0012] Kinshella, M. L. W. , Pickerill, K. , Bone, J. N. , Prasad, S. , Campbell, O. , Vidler, M. , Craik, R. , Volvert, M. L. , Mistry, H. D. , Tsigas, E. , Magee, L. A. , von Dadelszen, P. , Moore, S. E. , & Elango, R. , PRECISE Conceptual Framework Working Group . (2023). An evidence review and nutritional conceptual framework for pre‐eclampsia prevention. British Journal of Nutrition, 130(6), 1065–1076. 10.1017/S0007114522003889 36484095 PMC10442797

[mcn13722-bib-0013] Kiserud, T. , Piaggio, G. , Carroli, G. , Widmer, M. , Carvalho, J. , Neerup Jensen, L. , Giordano, D. , Cecatti, J. G. , Abdel Aleem, H. , Talegawkar, S. A. , Benachi, A. , Diemert, A. , Tshefu Kitoto, A. , Thinkhamrop, J. , Lumbiganon, P. , Tabor, A. , Kriplani, A. , Gonzalez Perez, R. , Hecher, K. , … Platt, L. D. (2017). The World Health Organization fetal growth charts: A multinational longitudinal study of ultrasound biometric measurements and estimated fetal weight. PLoS Medicine, 14(1), e1002220. 10.1371/journal.pmed.1002220 28118360 PMC5261648

[mcn13722-bib-0014] Kurpad, A. V. , & Sachdev, H. S. (2022). Precision in prescription: Multiple micronutrient supplements in pregnancy. The Lancet Global Health, 10(6), e780–e781. 10.1016/S2214-109X(22)00207-8 35561708 PMC7612989

[mcn13722-bib-0015] Mehrtash, H. , Stein, K. , Barreix, M. , Bohren, M. A. , & Tuncalp, O. (2023). Measuring women's experiences during antenatal care (ANC): Scoping review of measurement tools. Reproductive Health, 20(1), 150. 10.1186/s12978-023-01653-5 37817135 PMC10565981

[mcn13722-bib-0016] Mehrtash, H. , Stein, K. , Barreix, M. , Bonet, M. , Bohren, M. A. , & Tunçalp, Ö. (2023). Measuring women's experiences during antenatal care (ANC): Scoping review of measurement tools. Reproductive Health, 20(1), 150. 10.1186/s12978-023-01653-5 37817135 PMC10565981

[mcn13722-bib-0017] Napolitano, R. , Dhami, J. , Ohuma, E. , Ioannou, C. , Conde‐Agudelo, A. , Kennedy, S. , Villar, J. , & Papageorghiou, A. (2014). Pregnancy dating by fetal crown‐rump length: A systematic review of charts. BJOG: An International Journal of Obstetrics & Gynaecology, 121(5), 556–565. 10.1111/1471-0528.12478 24387345

[mcn13722-bib-0018] National Library of Medicine (U.S.) . (2024). *Multiple micronutrient supplementation for maternal anemia prevention in Tanzania (MMS‐MAP)*. Identifier NCT06079918. https://clinicaltrials.gov/study/NCT06079918

[mcn13722-bib-0019] Noguchi, L. , Bucagu, M. , & Tunçalp, Ö. (2023). Strengthening antenatal care services for all: Implementing imaging ultrasound before 24 weeks of pregnancy. BMJ Global Health, 8(5), e011170. 10.1136/bmjgh-2022-011170 PMC10255018 37257940

[mcn13722-bib-0020] Papageorghiou, A. T. , Kemp, B. , Stones, W. , Ohuma, E. O. , Kennedy, S. H. , Purwar, M. , Salomon, L. J. , Altman, D. G. , Noble, J. A. , Bertino, E. , Gravett, M. G. , Pang, R. , Cheikh Ismail, L. , Barros, F. C. , Lambert, A. , Jaffer, Y. A. , Victora, C. G. , Bhutta, Z. A. , & Villar, J. , International Fetal and Newborn Growth Consortium for the Century . (2016). Ultrasound‐based gestational‐age estimation in late pregnancy. Ultrasound in Obstetrics & Gynecology: The Official Journal of the International Society of Ultrasound in Obstetrics and Gynecology, 48(6), 719–726. 10.1002/uog.15894 26924421 PMC6680349

[mcn13722-bib-0021] Papageorghiou, A. T. , Kennedy, S. H. , Salomon, L. J. , Ohuma, E. O. , Cheikh Ismail, L. , Barros, F. C. , Lambert, A. , Carvalho, M. , Jaffer, Y. A. , Bertino, E. , Gravett, M. G. , Altman, D. G. , Purwar, M. , Noble, J. A. , Pang, R. , Victora, C. G. , Bhutta, Z. A. , Villar, J. , & International Fetal and Newborn Growth Consortium for the Century , (2014). International standards for early fetal size and pregnancy dating based on ultrasound measurement of crown–rump length in the first trimester of pregnancy. Ultrasound in Obstetrics & Gynecology: The Official Journal of the International Society of Ultrasound in Obstetrics and Gynecology, 44(6), 641–648. 10.1002/uog.13448 25044000 PMC4286014

[mcn13722-bib-0022] Schulze, K. J. , Mehra, S. , Shaikh, S. , Ali, H. , Shamim, A. A. , Wu, L. S. F. , Mitra, M. , Arguello, M. A. , Kmush, B. , Sungpuag, P. , Udomkesmelee, E. , Merrill, R. , Klemm, R. D. W. , Ullah, B. , Labrique, A. B. , West, Jr. K. P. , & Christian, P. (2019). Antenatal multiple micronutrient supplementation compared to iron‐folic acid affects micronutrient status but does not eliminate deficiencies in a randomized controlled trial among pregnant women of rural Bangladesh. The Journal of Nutrition, 149(7), 1260–1270. 10.1093/jn/nxz046 31006806 PMC6602890

[mcn13722-bib-0023] Smith, E. R. , He, S. , Klatt, K. C. , Barberio, M. D. , Rahnavard, A. , Azad, N. , Brandt, C. , Harker, B. , Hogan, E. , Kucherlapaty, P. , Moradian, D. , Gernand, A. D. , & Ahmadzia, H. K. (2021). Limited data exist to inform our basic understanding of micronutrient requirements in pregnancy. Science Advances, 7(43), eabj8016. 10.1126/sciadv.abj8016 34678054 PMC8535830

[mcn13722-bib-0024] Stevens, G. A. , Beal, T. , Mbuya, M. N. N. , Luo, H. , Neufeld, L. M. , Addo, O. Y. , Adu‐Afarwuah, S. , Alayón, S. , Bhutta, Z. , Brown, K. H. , Jefferds, M. E. , Engle‐Stone, R. , Fawzi, W. , Hess, S. Y. , Johnston, R. , Katz, J. , Krasevec, J. , McDonald, C. M. , Mei, Z. , … Young, M. F. (2022). Micronutrient deficiencies among preschool‐aged children and women of reproductive age worldwide: A pooled analysis of individual‐level data from population‐representative surveys. The Lancet Global Health, 10(11), e1590–e1599. 10.1016/S2214-109X(22)00367-9 36240826 PMC10918648

[mcn13722-bib-0025] Tuncalp, Ö. , Rogers, L. M. , Lawrie, T. A. , Barreix, M. , Peña‐Rosas, J. P. , Bucagu, M. , Neilson, J. , & Oladapo, O. T. (2020). WHO recommendations on antenatal nutrition: An update on multiple micronutrient supplements. BMJ Global Health, 5(7), e003375. 10.1136/bmjgh-2020-003375 PMC7394017 32732247

[mcn13722-bib-0026] UNICEF, UNU, WHO Study Team . (2002). Multiple micronutrient supplementation during pregnancy (MMSDP): Efficacy trials. University College London. https://mjmp.org/wp-content/uploads/2014/12/Multiple-Micronutrient-Supplementation-During-Pregnancy-MMSDP-Efficacy-Trials-WHO-UNICEF-UNU1.pdf

[mcn13722-bib-0027] Vogel, J. P. , Dowswell, T. , Lewin, S. , Bonet, M. , Hampson, L. , Kellie, F. , Portela, A. , Bucagu, M. , Norris, S. L. , Neilson, J. , Gülmezoglu, A. M. , & Oladapo, O. T. (2019). Developing and applying a ‘living guidelines’ approach to WHO recommendations on maternal and perinatal health. BMJ Global Health, 4(4), e001683. 10.1136/bmjgh-2019-001683 PMC6703290 31478014

[mcn13722-bib-0028] World Health Organization, United Nations University, & United Nations Children's Fund (‎UNICEF) . (1999). Composition of a multi‐micronutrient supplement to be used in pilot programmes among pregnant women in developing countries: Report of a United Nations Children's Fund (‎UNICEF)‎. World Health Organization (‎WHO)‎ and United Nations University workshop. UNICEF. https://iris.who.int/handle/10665/75358

[mcn13722-bib-0029] World Health Organization . (2016a). International statistical classification of diseases and related health problems 10th revision . https://icd.who.int/browse10/2019/en 10.2471/BLT.25.294650 PMC13531092 42683092

[mcn13722-bib-0030] World Health Organization . (2016b). WHO recommendations on antenatal care for a positive pregnancy experience: Web annexes . https://iris.who.int/bitstream/handle/10665/250796/9789241549912-webannexes-eng.pdf 28079998

[mcn13722-bib-0031] World Health Organization. (2016c). *WHO recommendations on antenatal care for a positive pregnancy experience*. http://www.who.int/publications/i/item/9789241549912 28079998

[mcn13722-bib-0032] World Health Organization . (2020). WHO antenatal care recommendations for a positive pregnancy experience: Nutritional interventions update: Multiple micronutrient supplements during pregnancy . https://www.who.int/publications/i/item/9789240007789 32783435

