# Supplementary material for: Use of multiple micronutrient supplementation integrated into routine antenatal care: A discussion of research priorities
Source: Matern Child Nutr. 2024 Oct 2;21(1):e13722. doi: 10.1111/mcn.13722 (PMC11650040; doi:10.1111/mcn.13722)
Supplement: Supplementary file 1 — Supporting information. [file MCN-21-e13722-s001.docx]

**Box 2** Recent re-analyses and trials evaluating use of MMS during pregnancy

| **Study** | **Population** | **Intervention groups** | **Outcomes** | **Findings** | **Limitations and comments** |
| --- | --- | --- | --- | --- | --- |
| **Re-analyses** | | | | | |
| Keats 2022  Systematic review and meta-analysis of 13 RCTs | Pregnant adolescents (10-19 years of age) | MMS  IFA | -birth weight  - low birth weight (LBW)  -gestational age (GA)  -preterm birth (PTB)  -small for gestational age (SGA)  -stillbirth  -perinatal mortality  -neonatal mortality  -maternal hemoglobin level  -maternal anemia | In adolescents, MMS reduced LBW, preterm birth and SGA when compared to IFA | -The sample of younger adolescents (10–14 years) was too small to perform meaningful effect modification within this group or to compare younger adolescents with older adolescents  -It may be practically challenging to conduct studies exclusively targeting this group of pregnant girls aged 10 to 14 years. |
| Liu 2022  Two stage Meta-analysis of individual participant data of 14 trials | Pregnant women | MMS IFA  LNS | -Gestational weight gain (GWG) during pregnancy  -severely inadequate and excessive percentage of inadequate GWG  -no difference in excessive GWG  -adequacy of GWG  -higher GWG at delivery  - reduced severely (>125%) GWG | MMS was associated with greater GWG percentage adequacy and total GWG, and reduced severely inadequate GWG at delivery compared to IFA | A direct  measure of GWG during the entire pregnancy period was not always available, but a validated statistical modeling approach was used to estimate early pregnancy weight |
| Gomes 2023 (MMS TAG)  Meta-analysis of all trials from the Keats 2019 (Cochrane review) that assessed third trimester maternal anemia | Pregnant women | MMS  IFA  (subgroup analyses according to doses of iron in IFA and MMS) | - third trimester maternal anemia - hemoglobin concentration - iron deficiency anemia | Compared to IFA, MMS resulted in comparable hemoglobin concentration and protection against anemia during pregnancy, independently of iron dose. | For the main comparison of interest (MMS with 30 mg of iron versus IFA with 60 mg of iron), while a significant number of studies and participants (4677) contributed to the effect estimates of anemia, less data were available for the effect estimates of iron deficiency anemia. |
| Gomes  2023 (MMS TAG)  Meta-analysis of 13 trials included in WHO GDG analysis (all trials that assessed neonatal mortality) | Pregnant women | MMS  IFA (different doses of iron from 20 to 60 mg) | Neonatal mortality | Neonatal mortality did not differ between MMS and IFA regardless of iron dose in either supplement. | Wide confidence interval for analysis of 60 mg of iron vs MMS with 30 mg of iron (RR 1.12, 95 % CI 0.83 to 1.50). |
| Gomes  2023 (MMS TAG)  Meta-analysis of 16 trials included in WHO GDG analysis (all trials that assessed birth outcomes) | Pregnant women | MMS IFA | -birth outcomes by gestational age assessment method   - LBW - PTB - SGA | The effects of MMS, compared to IFA, on birthweight, preterm birth and SGA appeared consistent across subgroups with no evidence of subgroup differences. When limited to the seven trials that used ultrasound, beneficial effects of MMS were observed for LBW (risk ratio (RR 0.87, 95% CI 0.78-0.97), preterm birth (RR 0.90, (95% CI 0.79-1.03), and SGA (RR 0.9, 95% CI 0.83-0.99). | The meta-analysis followed the methodology of the WHO GDG analysis (included the same number of trials, used the same effect estimates in each study arm of each included trial, and did a sensitivity analysis limited to the trials that used UNIMMAP supplements in the intervention arm). |
| **Observational studies** | | | | | |
| Caniglia 2022 | Pregnant women in Botswana  (22.5% living with HIV) | MMS  IFA  Iron alone  Folic acid alone | - stillbirth - PTB - very PTB - SGA - very SGA - neonatal death - stillbirth or neonatal death - LBW - very LBW - third trimester anaemia - caesarean delivery - short length for GA | Compared with IFA, women who initiated MMS had lower risks of preterm and very preterm births, and low and very low birthweight | -The study had information on the first supplement that was prescribed and filled, but not on subsequent prescriptions or on adherence.  -Women in this study (a real-world setting) generally initiated supplementation later in pregnancy than many women enrolled in the trials and therefore, it is possible that the duration of supplementation did not reach an adequate threshold to have an effect on certain outcomes, such as SGA |
| **New trials** | | | | | |
| Bliznashka 2022 | Pregnant women in rural Niger- enrolled in live rota virus vaccine study | MMS IFA  Lipid-based nutrient supplementation (LNS) | -child length for age z scores  -weight for age z scores  -weight for length z scores at 24 months | Prenatal MMS had limited effect on anthropometric measures of child growth up to 24 months of age compared to IFA | -Lack of data on maternal biomarkers and therefore unable to directly assess intermediate effects of the study supplements on maternal micronutrient status  -Lack of data on maternal infection and could not determine the extent to which maternal infection may have influenced nutrient availability  -Child growth was only assessed through anthropometry rather than body composition |

**References**

Keats, E. C., Akseer, N., Thurairajah, P., Cousens, S., Bhutta, Z. A., & the Global Young Women’s Nutrition Investigators’ Group. (2022). Multiple-micronutrient supplementation in pregnant adolescents in low-and middle-income countries: a systematic review and a meta-analysis of individual participant data. *Nutrition Reviews, 80*(2), 141-156. <https://doi.org/10.1093/nutrit/nuab004>

Liu, E., Wang, D., Darling, A. M., Perumal, N., Wang, M., Ahmed, T., Christian, P., Dewey, K. G., Kac, G., Kennedy, S., Subramoney, V., Briggs, B., Fawzi, W. W., & members of the GWG Pooling Project Consortium. (2022). Effects of prenatal nutritional supplements on gestational weight gain in low-and middle-income countries: a meta-analysis of individual participant data. *The American journal of clinical nutrition, 116*(6), 1864-1876. <https://doi.org/10.1093/ajcn/nqac259>

Bottom of Form

Gomes, F., Agustina, R., Black, R. E., Christian, P., Dewey, K. G., Kraemer, K., Shankar, A. H., Smith, E. R., Thorne-Lyman, A., Tumilowicz, A., & Bourassa, M. W. (2022). Multiple micronutrient supplements versus iron‐folic acid supplements and maternal anemia outcomes: An iron dose analysis. *Annals of the New York Academy of Sciences, 1512*(1), 114-125. <https://doi.org/10.1111/nyas.14756>

Gomes, F., Agustina, R., Black, R.E., Christian, P., Dewey, K.G., Kraemer, K., Shankar, A.H., Smith, E., Tumilowicz, A., & Bourassa, M.W. (2022). Effect of multiple micronutrient supplements vs iron and folic acid supplements on neonatal mortality: a reanalysis by iron dose. *Public Health Nutrition, 25*(8), 1-13. https://doi.org/10.1017/S1368980022001008

Gomes, F., Askari, S., Black, R. E., Christian, P., Dewey, K. G., Mwangi, M. N., Rana, Z., Reed, S., Shankar, A. H., Smith, E. R., & Tumilowicz, A. (2023). Antenatal multiple micronutrient supplements versus iron‐folic acid supplements and birth outcomes: Analysis by gestational age assessment method. *Maternal & Child Nutrition*, e13509. <https://doi.org/10.1111/mcn.13509>

Caniglia, E. C., Zash, R., Swanson, S. A., Smith, E., Sudfeld, C., Finkelstein, J. L., Diseko, M., Mayondi, G., Mmalane, M., Makhema, J., Fawzi W., Lockman, S., & Shapiro, R. L. (2022). Iron, folic acid, and multiple micronutrient supplementation strategies during pregnancy and adverse birth outcomes in Botswana. *The Lancet Global Health, 10*(6), e850-e861. <https://doi.org/10.1016/S2214-109X(22)00126-7>

Bliznashka, L., Sudfeld, C. R., Garba, S., Guindo, O., Soumana, I., Adehossi, I., Langendorf, C., Grais, R. F., & Isanaka, S. (2022). Prenatal supplementation with multiple micronutrient supplements or medium-quantity lipid-based nutrient supplements has limited effects on child growth up to 24 months in rural Niger: a secondary analysis of a cluster randomized trial. *The American journal of clinical nutrition, 115*(3), 738-748. <https://doi.org/10.1093/ajcn/nqab404>

**Table 1 Studies examining biological impact pathways of MMS during pregnancy**

| **Study** | **Country** | **Intervention and comparison groups** | **Potential pathway** | **Findings** |  |
| --- | --- | --- | --- | --- | --- |
| Chen (2019) | China | MMS  IFA | Hypertensive Disorders of Pregnancy | -There were no significant differences in prevalence of pregnancy-induced hypertension (PIH) across supplement groups  -There was no difference in prevalence of severe or early onset PIH across supplement groups  -Among women whose hemoglobin level was greater than 13.2 g/dL, MMS had a significant reduced odds of PIH compared with either FA or compared with IFA  **-**The incidence of PIH was statistically significantly lower among women who began MMS supplementation before 12 weeks compared with women who began MMS supplementation at 12 weeks or later  -A similar protective effect was observed for both early-onset and late-onset of PIH  -Chronic hypertension and pre-eclampsia were not assessed | |
| Christian (2006) | Nepal | IFA + vitamin A  Iron + zinc + vitamin A  Folic acid + vitamin A  MMS + vitamin A  Vitamin A alone | Infection | -The baseline mean blood concentrations of micronutrients and markers of subclinical infection CRP [C reactive protein] and AGP [alpha 1-acid glycoprotein] did not differ by treatment group  -Subclinical infection was not defined  -No pregnancy specific range for CRP has been established  -It is not known what the cut-off for CRP is in sub-clinical infections in pregnant women | |
| Christian (2016) | Nepal | IFA + vitamin A  Iron + zinc + vitamin A  Folic acid + vitamin A  MMS + vitamin A  Vitamin A alone | - Stress (cortisol) - anaemia related hypoxia (erythropoietin/EPO) | Although this study did not analyze the association of hormone levels and birth outcomes by supplementation group, it found:   - Cortisol concentrations did not differ in any of the supplement groups - Cortisol was negatively associated with length of gestation and a higher risk of preterm birth - Circulating concentrations of EPO were significantly lower in each of the groups that included iron in the supplement formulation - Third trimester EPO was associated with a reduction in low birthweight | |
| Gernand (2015) | Bangladesh | MMS  IFA | Fetal growth factors   - insulin-like growth factor (IGF) - human placental lactogen (hPL) - placental growth hormone (PGH) | No difference in mean concentrations of hPL, PGH, insulin, IGF-1 or IGFBP-1 at 32 weeks between those receiving MMS compared to IFA   - While JiViTA found significant reductions in pre-term birth and low birth weight in the MMS group, in this proportionally small subgroup of the parent trial, those differences in birth outcomes were not reflected | |
| Hindle (2006) | Nepal | MMS  IFA | Inflammation | -Blood eosinophils, plasma concentrations of the acute phase reactants and the production of interleukin (IL) 10, IL-4, interferon, and tumor necrosis factor did not differ significantly between the groups  -Plasma CRP and AGP were higher in women who had a preterm delivery, and AGP was higher in women who delivered a low-birth-weight term infant than in women who delivered a normal-birth-weight term infant  -32 weeks chosen as cut off for pre-term delivery   - -The association of inflammatory markers and adverse birth outcomes were not analyzed by supplementation groups | |
| Hininger (2004) | France | Placebo  MMS | Oxidative stress | - No statistical differences in oxidative stress parameters between placebo and MMS groups - Both groups had higher than average vitamin C levels but placebo group had lower baseline vitamin C levels compared to MMS groups - LBW mediated by other effects of vitamin C other than oxidative stress | |
| Liu (2021) | China | MMS  IFA | Hypertensive Disorders of Pregnancy | -There were no significant differences in prevalence of pregnancy-induced hypertension (PIH) across supplement groups  -There was no difference in prevalence of severe or early onset PIH across supplement groups  -Among women whose hemoglobin level was greater than 13.2 g/dL, MMS had a significant reduced odds of PIH compared with either FA or compared with IFA  **-**The incidence of PIH was statistically significantly lower among women who began MMS supplementation before 12 weeks compared with women who began MMS supplementation at 12 weeks or later  -A similar protective effect was observed for both early-onset and late-onset of PIH  -Chronic hypertension and pre-eclampsia were not assessed | |
| Priliani (2018 and 2019) | Indonesia | MMS IFA | Mitochondrial DNA copy number [mtDNA-CN] | Although mtDNA-CN in the two groups were not correlated to birth outcomes in this study, it found:   - Higher maternal mtDNA-CN at enrollment was associated with a 204.6 g decrease in birth weight - In both groups, maternal mtDNA-CN at post-supplementation was significantly elevated compared with baseline - The MMS group had lower posts-supplementation mtDNA-CN than the IFA group   This study did not find a difference in supplement effect in anemic women, which was felt to be possibly due to the modest sample size  The findings should be interpreted in the context of the parent trial SUMMIT where MMS supplementation reduced fetal loss and early infant mortality and improved birth weight particularly in anemic women | |

References

Hininger, I., Favier, M., Arnaud, J., Faure, H., Thoulon, J. M., Hariveau, E., Favier, A., & Roussel, A. M. (2004). Effects of a combined micronutrient supplementation on maternal biological status and newborn anthropometrics measurements: a randomized double-blind, placebo-controlled trial in apparently healthy pregnant women. *European journal of clinical nutrition, 58*(1), 52-59. <https://doi.org/10.1038/sj.ejcn.1601745>

Christian, P., Nanayakkara‐Bind, A., Schulze, K., Wu, L., LeClerq, S. C., & Khatry, S. K. (2016). Antenatal micronutrient supplementation and third trimester cortisol and erythropoietin concentrations*. Maternal & child nutrition, 12*(1), 64-73. <https://doi.org/10.1111/mcn.12138>

Priliani, L., Febinia, C. A., Kamal, B., Shankar, A. H., & Malik, S. G. (2018). Increased mitochondrial DNA copy number in maternal peripheral blood is associated with low birth weight in Lombok, Indonesia. *Placenta, 70*, 1-3. <https://doi.org/10.1016/j.placenta.2018.08.001>

Priliani, L., Prado, E. L., Restuadi, R., Waturangi, D. E., Shankar, A. H., & Malik, S. G. (2019). Maternal multiple micronutrient supplementation stabilizes mitochondrial DNA copy number in pregnant women in Lombok, Indonesia. *The Journal of nutrition, 149(*8), 1309-1316. <https://doi.org/10.1093/jn/nxz064>

Gernand, A. D., Schulze, K. J., Nanayakkara-Bind, A., Arguello, M., Shamim, A. A., Ali, H., Wu, L., West Jr., K. P., & Christian, P. (2015). Effects of prenatal multiple micronutrient supplementation on fetal growth factors: a cluster-randomized, controlled trial in rural Bangladesh. *PLoS One, 10*(10), e0137269. <https://doi.org/10.1371/journal.pone.0137269>

Chen, S., Li, N., Mei, Z., Ye, R., Li, Z., Liu, J., & Serdula, M. K. (2019). Micronutrient supplementation during pregnancy and the risk of pregnancy-induced hypertension: A randomized clinical trial. *Clinical nutrition, 38*(1), 146-151. <https://doi.org/10.1016/j.clnu.2018.01.029>

Liu, Y., Li, N., Mei, Z., Li, Z., Ye, R., Zhang, L., Li, H., Zhang, Y., Liu, J., & Serdula, M. K. (2021). Effects of prenatal micronutrients supplementation timing on pregnancy‐induced hypertension: Secondary analysis of a double‐blind randomized controlled trial. *Maternal & Child Nutrition, 17*(3), e13157. <https://doi.org/10.1111/mcn.13157>

Christian, P., Jiang, T., Khatry, S. K., LeClerq, S. C., Shrestha, S. R., & West Jr, K. P. (2006). Antenatal supplementation with micronutrients and biochemical indicators of status and subclinical infection in rural Nepal. *The American journal of clinical nutrition, 83*(4), 788-794. <https://doi.org/10.1093/ajcn/83.4.788>

Hindle, L. J., Gitau, R., Filteau, S. M., Newens, K. J., Osrin, D., Costello, A. M., Anjana, V., Kumar, M. R., Birendra, Y., & Manandhar, D. S. (2006). Effect of multiple micronutrient supplementation during pregnancy on inflammatory markers in Nepalese women. *The American journal of clinical nutrition, 84*(5), 1086-1092. <https://doi.org/10.1093/ajcn/84.5.1086>
